# Stability Analysis of Plate—Screw Fixation for Femoral Midshaft Fractures

**DOI:** 10.3390/ma16175958

**Published:** 2023-08-30

**Authors:** Izzawati Basirom, Ruslizam Daud, Muhammad Farzik Ijaz, Mohd Afendi Rojan, Khairul Salleh Basaruddin

**Affiliations:** 1 Fracture and Damage Mechanics (FDM), Faculty of Mechanical Engineering Technology, University Malaysia Perlis, Arau 02600, Perlis, Malaysia; izzawati.basirom@gmail.com (I.B.); ruslizam@unimap.edu.my (R.D.); afendirojan@unimap.edu.my (M.A.R.); khsalleh@unimap.edu.my (K.S.B.); 2 Mechanical Engineering Department, College of Engineering, King Saud University, P.O. Box 800, Riyadh 11421, Saudi Arabia

**Keywords:** Implant stability, interfragmentary strain, transverse fracture, screw configuration

## Abstract

An understanding of the biomechanical characteristics and configuration of flexible and locked plating in order to provide balance stability and flexibility of implant fixation will help to construct and promote fast bone healing. The relationship between applied loading and implantation configuration for best bone healing is still under debate. This study aims to investigate the relationship between implant strength, working length, and interfragmentary strain (εIFM) on implant stability for femoral midshaft transverse fractures. The transverse fracture was fixed with a fragment locking compression plate (LCP) system. Finite element analysis was performed and subsequently characterised based on compression loading (600 N up to 900 N) and screw designs (conventional and locking) with different penetration depths (unicortical and bicortical). Strain theory was used to evaluate the stability of the model. The correlation of screw configuration with screw type shows a unicortical depth for both types (*p* < 0.01) for 700 N and 800 N loads and *(p* < 0.05) for configurations 134 and 124. Interfragmentary strain affected only the 600 N load (*p* < 0.01) for the bicortical conventional type (group BC), and the screw configurations that were influenced were 1234 and 123 (*p* < 0.05). The low steepness of the slope indicates the least εIFM for the corresponding biomechanical characteristic in good-quality stability. A strain value of ≤2% promotes callus formation and is classified as absolute stability, which is the minimum required value for the induction of callus and the maximum value that allows bony bridging. The outcomes have provided the correlation of screw configuration in femoral midshaft transverse fracture implantation which is important to promote essential primary stability.

## 1. Introduction

Orthopaedic bridge-plating fixation has been studied extensively. Bone-healing studies have highlighted the complications involved in the delayed union, non-unions, fracture after device removal, and infection, mostly in the diaphysis and meta-diaphyseal fractures [1,2]. Subsequently, the complication risks were minimised by utilising biomechanical behaviour knowledge of the implant fixation and tissue vascularity. In recent years, the development of improved implant fixation has provided reliable stability for orthopaedic surgeon applications and has been of considerable interest [3,4,5]. Current biomechanical improvement efforts have been focused on to enhance the bone–intramedullary nail system stability [6]; for example, the exploration of using additive manufacturing and the finite element (FE) method for the design, diagnosis, and planning of a locked intramedullary nail used in the diaphyseal femoral fractures mainly to simulate the osseointegration bone implant [7]. Based on statistical data, implant failure that occurred on the plate (42.4%) was more common than unlocked nails (33.3%) and subsequently locked nails (24.2%); occurrences of this failure were reported that were the result of fatigue failure (42.4%), bending (27.27%), loosening (18.8%), and infection (5.5%) [8]. In addition, researchers also revealed that implant failure was caused by the stress-shielding effect and overloading condition in the implant [9,10]. Subsequently, implant loosening may influence the bone remodelling process, whereas bone loss around screws leads to fixation failure. Thus, it is essential to consider the implant stability and the critical stress of implant fixation.

Among all the implant fixations, the locking compression plate was improved to achieve better fracture union compared with conventional plating which was stiffer in allowing bone healing. Moreover, flexibilities of the locking characteristic were necessary to enable interfragmentary motion (IFM) to directly stimulate a new bone bridge at the fracture site [11]. The rigid engagement of the locking screw to the plate provides fixed-angle stabilisation compared with the plate–bone compression of the conventional screw [12]. A few studies suggested that fixed-angle stabilisation with a unicortical locking screw provides distinct advantages over the conventional screw. However, the advantages remain inadequate for both screw types.

The function of a bridge plating with a screw construct was developed in advance based on a stability requirement of a particular fracture. These implants act as load-sharing or load-bearing devices, depending on the fracture reduction and fragment interference [13]. Implant instability and mechanical failure are related to poor stress-sharing loads and implant loosening [14,15]. Despite the high flexural strength, implant fixation can distribute the induced stresses. The effect of the screw types and configuration on the stress sharing, however, has been mentioned less. In addition, implant stability is influenced by such factors as the type of loading, the implant connection, the type of material, and the bone–implant interfaces [16,17,18,19]. It is necessary to analyse the bone properties, structure, and load resistance in biomechanics analysis [20]. A similar factor to be considered is the implant contact pressure between the femur and tibia [20]. To date, the finite element (FE) approach that employs structural analysis is the most successful approach for simulating the bone–implant interface.

Different designs of bone geometry have shown different results for stress and subsequent deformations. Two-dimensional FE modelling representing the bone–implant structure is based on the assumption that the load is axially symmetrical [21]. Most of the 2D-model studies give satisfactory insight into the behaviour of the bone–implant interface. However, the stress analysis of bone predicted by the 2D model is also known to be less accurate compared with the 3D model [22,23]. This is because the assumptions of the 2D model only consider a cross section view to represent the information in bone tissue around the implant. However, a 3D-modelling technique must be adopted first to determine the external loading in order to show the overall deformation and stress distribution.

In one study, the stability spectrum was scaled as a quantitative condition of implant stability [24]. Micromotion is essential to improve the osseointegration processes in broken bones and indirectly provides biological or secondary stability [25,26,27,28,29]. However, excessive movements can reduce the stability of the implant and the healing of the bones. Therefore, knowledge of both primary and secondary stability and the spectrum is emphasised in this study.

Some studies suggest that optimal screw configuration can minimise healing time and also surgical costs [30]. Researchers established that the number of screws increased the rigidity of the implant structure. Instead, one fundamental concept found throughout the literature is that more screws do not mean more stability [31]. It can be concluded that, for a diaphysis fracture, three or four screws are sufficient [32]. This study aims to evaluate the mechanical behaviour of a fragment locking compression plate (LCP) system as a function of screw type, screw configuration, and penetration depth using a finite element model (FEM). The literature on the subject has contrasting recommendations on optimal screw numbers and configurations, mostly based on empirical evidence.

## 2. Materials and Methods

### 2.1. Implant–Bone Fixation System

**Figure 1:**
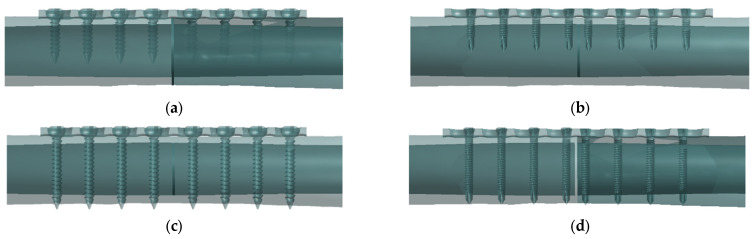
Configuration of plate–screw implant–bone fixation: (**a**) unicortical conventional (UC), (**b**) unicortical locking (UL), (**c**) bicortical conventional (BC), and (**d**) bicortical locking (BL).

In this study, the three stages of internal implant fixation are involved which are implant–bone fixation, implant–bone loading condition, and implant–bone screw penetration. Implant fixation was studied based on the mechanical response: (i) when implant placement is performed on the femur, (ii) when the compression loading is applied on the femur bone, and (iii) there is a different type of screw and penetration depth. The four surgical fixation groups—unicortical conventional screw (UC), unicortical locking screw (UL), conventional bicortical screw (BC), and bicortical locking screw (BL)—are shown in Figure 1.

### 2.2. 3D Model of Implant–Bone Configuration

**Figure 2:**
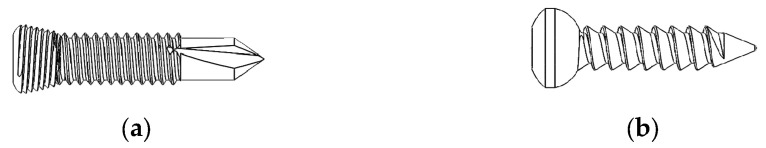
Types of plating screws: (**a**) locking screw and (**b**) conventional screw.

**Figure 3:**
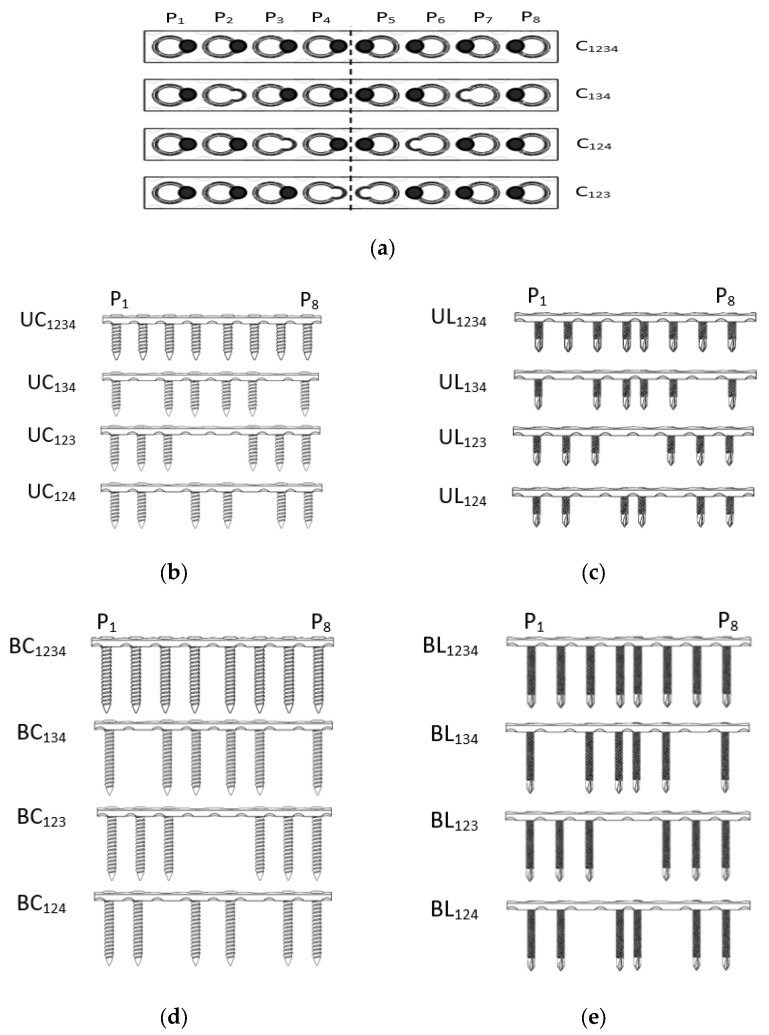
Screw configurations based on implant group (**a**) screw position, (**b**) unicortical conventional (UCn), (**c**) unicortical locking (ULn), (**d**) bicortical conventional (BCn), and (**e**) bicortical locking (BLn) where n is the screw configuration.

Three parts of the fixation system were constructed: screws, a compression plate, and an intact femur bone. The intact femur model was applied before mimicking the actual bone from CT scan through the computer-aided design (CAD) model library [33]. The models were a modified cortex thickness averaging approximately 5 mm in thickness, and the midsection of the femur bone was the focus. The screw and a fragment compression plate were designed using SolidWorks version 2020, Mechanical Design Lab, Faculty of Mechanical Engineering Technology (Dassault, MA, USA). All implants were designed according to the Synthes product [34]. Reverse engineering has been defined for obtaining the geometry of implant parts. An adaptive reconstruction method used by enclosing the original point creates a point set by recursively subdividing each point into a sub-point. In addition, the resulting mesh is obtained by subdividing the coarser mesh and adapting the topology at the location where points have been removed. Final mapping locally constraining the mesh toward the concentrated point will often influence accuracy and results. In this study, conventional screw lengths of 18 mm (product number: 204.818) and 36 mm (product number: 204.821) were used. Subsequently, locking screws of 18 mm (product number: 212.112) and 36 mm (product number: 212.105) and a compression plate with eight holes were used (product number: 223.581). Both types of screw models were designed as shown in Figure 2. ANSYS Design Modular v18 was used to assemble the bone and implant using Boolean operation. In this study, the static structural module was used to simulate the bone implant fixation. Figure 3a shows that eight holes in the LCP were numbered from Position 1 to 8 (P_1_–P_8_), and the four configurations are shown in Figure 3b–e.

In accordance with the Arbeitsgemeinschaft für Osteosynthesefragen/Association of the Study of Internal Fixation (AO/ASIF) procedure [35], the plate is located on the lateral side of the femur. This study involved the locking conventional screw construction with LCP. Four groups were categorized—group UC, group UL, group BC, and group BL—and then followed by the number of configurations. These configurations were selected as the control group based on current configurations in clinical practice [31]. A 1 mm fracture gap was used between bone fragments as suggested by the previous study that the gap should not exceed 2 mm for osseointegration [36].

### 2.3. Mesh and Validation

**Figure 4:**
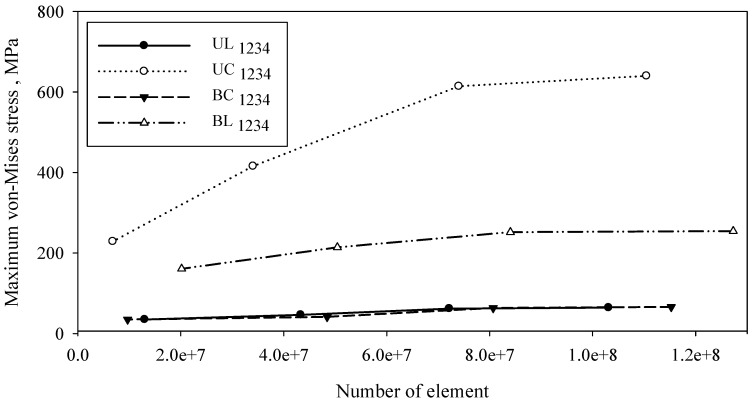
Effect of element number on the von-Mises stress based on different screw types of C_1234_.

**Figure 5:**
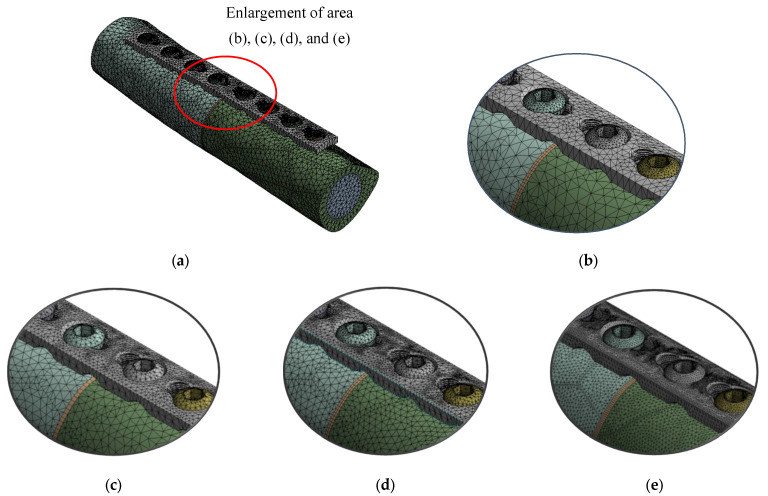
Meshed model with the enlargement area. (**a**) Meshed implant model (1.0 mm), (**b**) default, (**c**) 1.0 mm (coarse), (**d**) 0.6 mm (medium), (**e**) 0.4 mm (fine).

Tetrahedron element mesh was used for all models. Table 1 shows the statistics of mesh with nodes and elements of the level of element sizes for different types of implant configuration. The model validation is based on internal FE validation through convergence analysis. Figure 4 shows a convergence test performed for 16 types of configuration according to groups UC, UL, BC, and BU. Figure 5 shows the meshed model with an enlargement area for four variations of mesh: default, coarse, medium, and fine size. An element size of 0.4 mm with a transition ratio of 0.272 and a growth rate of 1.2 was used.

### 2.4. Fixation Interface Condition

The interaction approach between contact pair is classified as bone–screw, implant–screw, and implant–bone. The fixation system model employed the interaction as a simple contact that was frictionless between the implant and bone surface. The friction coefficients between 0.1 and 0.3 were compared [24], and the result shows that friction has a less significant effect on bone–implant stress results [37]. The implant is assembled as a model of tightening to define it in such a way that it describes complete bone healing surrounding the implant. Despite the minimal effect of friction on the result, it can be clarified as a rigid or contact pair between bone–implant interfaces. The different interaction properties are summarised in Table 2.

### 2.5. FE Simulation of Compression Load

**Figure 6:**
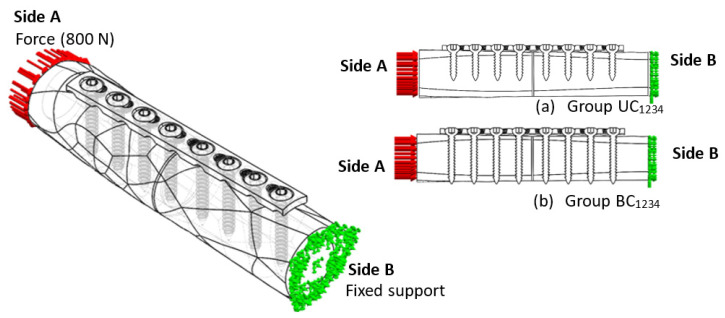
Boundary condition of compression load: (**a**) unicortical and (**b**) bicortical conventional screw setup.

A constant load of 800 N was applied once the preliminary steps were achieved. It has been demonstrated to correspond to the load on the femur during regular activities such as standing up. The rationale relation between dynamic and static loading demonstrated that the average dynamic force applied on the femur is representative of the constant load based on human weight (i.e., 600 N, 700 N, 800 N, 900 N). Sides A and B represent the compression loading and constraint, respectively, and the schematic of these four loading conditions is illustrated in Figure 6a,b. This assumption approximates the actual condition of the anterior and posterior regions of the femur shaft. A simplified model was used to provide information about the screw–plate configuration affecting the mechanical behaviour of the bone–implant system [38]. This simplified model allows the parametric study of bone–implants involving stress distribution and stress shielding that influence the construct’s stability. The assembly force used in this study was applied as a compression rather than an impact, as is the case during surgery. A dynamic impact load can be replaced by compression as it results in a similar strength [39].

### 2.6. Identification of Material Properties

Table 3 shows the mechanical properties of the bone–implant model. The femur bone consists of two sections: cortical and trabecular bone. The orthotropic elastic behaviour was used for the cortical bone, whereas the other parts are assumed to behave as the isotropic elastic behaviour. Orthotropic elasticity can represent actual bone properties because it can show the bone structure composition [40,41]. Additionally, simplified biological information was used, such as a 1% formed callus representative of 0.2 GPa of Young’s modulus in the 1 mm fracture gap. The compression plate and screw parts and their material properties were measured [42].

### 2.7. Stability Characterisation

The primary stability of implant fixation is related to the implant’s micromotion, which is defined as a displacement that refers to a small movement of bone fragments at the fracture site. It can be characterised by the implant’s stress analysis, working length analysis, interfragmentary strain measurement, and stability classification.

#### 2.7.1. Implant Stress Analysis

The implant fixation with two screw types was modelled; subsequently, the implant strength was appropriately computed. The four screw types were simulated by four configurations at various penetration depths. The overall results were determined using the maximum von Mises stress of the implant. The mechanical properties of stainless steel were used to estimate the stress required to initiate the crack and to cause implant loosening and failure. The changing stress value affects Young’s modulus bone neighbour and causes jumps in stress values in the bone.

#### 2.7.2. Working Length Measurement

Further investigation was carried out in simulating a relationship between working length and maximum von Mises stress by taking the average of the implant equivalent of stress for each working length. Working length refers to the distance between the two innermost screws. By omitting one screw hole on either side of the fracture, the construct became flexible in both compression and torsion. The relationship between working length and implant stress was determined, in order to differentiate the rigidity and flexibility based on screw configurations.

#### 2.7.3. Interfragmentary Strain Measurement

Interfragmentary strain at the fracture gap filled by the callus was calculated using the comparison of the initial length of the fracture gap and the gap size under stress based on the end point of both bone fragment connectors with the callus. Perren’s strain theory was used for observing the implant stability. Strain values up to 2% promoted the formation of lamella tissue and at this stage can be classified as absolute stability. In addition, up to 10% are tolerated by the woven bone formation, and for between 10% and 30%, induction of resorption prevails. The interfragmentary of each construct was determined using the following equation:

(1) $$\varepsilon_{IF}=∆L/L$$

where $\varepsilon_{IF}$ is interfragmentary strain, ∆*L* is changing between initial length and length under stress, and *L* is the initial length = 1 mm.

#### 2.7.4. Stability Classification

Implant stability was evaluated based on screw type and its configuration. Absolute stability was achieved by compression load at the fracture site, where small gaps of 1 mm are bridged by bone growth. The stabilisation at the fracture site determined the further course of bone healing [43]. Stability is divided into three scales: absolute stability (2%), relative stability (range 2% to 10%), and instability (greater than 10%); interfragmentary strain can be utilised to account for bone healing rate. The stability of interfragmentary strain was determined using the following conditions:

(2) $$\varepsilon_{IF}\leq 2\%\;\;\;\;\;\mathrm{Absolute}\;\mathrm{stability}$$

(3) $$2\%\leq \varepsilon_{IF}\leq 10\%\;\;\mathrm{Relative}\;\mathrm{stability}$$

(4) $$\varepsilon_{IF}\leq 10\%\;\;\mathrm{Instability}$$

## 3. Results and Discussion

### 3.1. Effect of Screw Types and Penetration Depth Configuration

**Figure 7:**
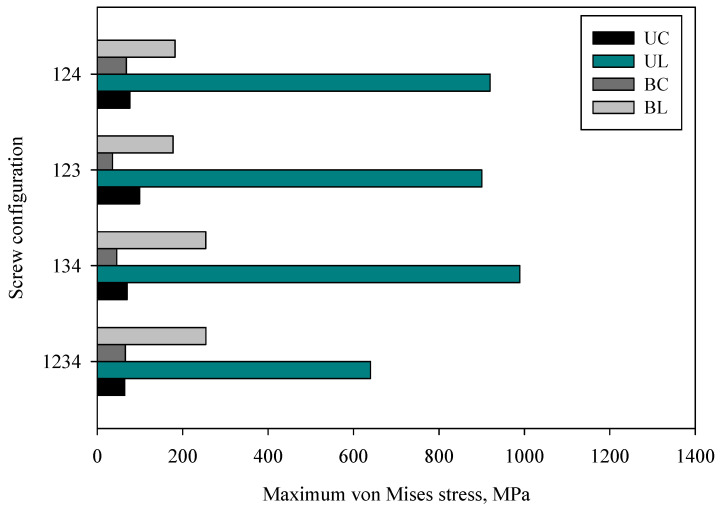
The effect of screw configuration on the implant stress.

Figure 7 shows the effects of screw configuration on the implant stress for different screw types. In general, the different screw types and configurations yield a different stress distribution threshold. The results obtained for the Groups UL and BL were superior to those of Groups UC and BC. The implant strengths of Groups UC and BC were relatively lower and uniform. On the contrary, the strength of Groups UL and BL was rough and irregular. As the screw configurations changed, the implant strengths of both penetration depths were reduced, indicating optimal stability for bone–implant fixation.

When the configurations of screw types were compared, group UC showed the highest strength of UC_123_ with a maximum of 99.24 MPa, followed by the UC_124_, which had a maximum stress of 76.78 MPa. The most stable construct was observed for UC_1234_, with a minimum stress of 64.48 MPa; moreover, this configuration had considerably larger stability than those seen for UC_134_, UC_123_, and UC_124_. The high stress of the implant leads to mechanical failure. However, the low stresses may cause stress shielding, which could lead to bone resorption and implant loosening [44].

Considering the locking screw construction with various screw configurations, all exhibit high values concerning the penetration depth. The maximum strength (989.26 MPa) was determined in group UL_134_. Groups UL_1234_, UL_123_, and UL_124_ yielding average strengths of 639.77 MPa, 900.65 MPa, and 919.59 MPa, respectively. The implant strength of Groups UC and UL was relatively higher for Configuration 1234. Subsequently, UC_1234_ shows the least stress value compared with UL_1234_. This is due to the exceeding resistive strength of cortical bone, whereas bone resorption and screw loosening occurred. This loosening occurred because of the high interfragmentary strain, which can increase micro movement at the fracture gap and subsequently change the implant stability level [45].

Compared with group UL, critical stress was found at UL_134_ with a maximum of 989.26 MPa, followed by UL_124_, which had maximum stress of 919.59 MPa. The most stable group UL construct was found to be UL_1234_, which had a maximum stress value of 639.77 MPa. In hindsight, group UC and group UL for C1234 were the most stable, while the UC_1234_ construct yielded lower stress than UL_1234_. This is because of an axial load exceeding the frictional force and producing a high-stress transfer to the bones and neighbouring interfaces.

Group UL_123_ showed the least strength (177.75 MPa), while the configurations of UL_1234_, UL_134_, and UL_124_ had strengths of 254.41 MPa, 254.23 MPa, and 182 MPa, respectively. In addition, for group BL, BL_123_ experienced the maximum stress of 99.24 MPa, which is similar to the stress experienced by the BL_1234_ (64.48 MPa), BL_134_ (70.12 MPa), and BL_123_ (76.78 MPa) configurations. Groups BC and BL exhibit that C_123_ was the most stable. This attests to an earlier suggestion that the number of screws does not necessarily equate to an increase in stability. The fewer-screw predictably will increase the load sharing for individual screws; however, the stability also can be controlled by changing the working length. A similar observation was also reported by Stoffel [46].

Groups BC and BL were very stable compared with groups UC and UL because both groups penetrate through the two stiff layers of the bone cortex. Shortening of the screw length results in a substantial increase in the compressive stress [14], whereas the screw diameter influenced the strength or holding power [35]. The load sharing to the bone was higher for the conventional screw compared with the locking screw. The stress distributes from the plate to the screw head through locking threads. This is caused by the high wear rate for the bone, as the bone cortex creates counteracting forces under axial loading, mainly at the implant interface. These changing stress values affect the consistency of Young’s modulus bone neighbouring and cause jumps in stress values in the bone. Thus, major jumps in stress occur in bone. The reason behind these jumps is the nature of bone remodelling and changing values of Young’s modulus bone during the healing process. The highlighted finding was that the high stress of implants provides a less stable bone–implant system. This can be observed from group UC_123_ (35.78 MPa) being the most stable group. The strength and deformation of the implant depend on the bending moment of plating the bridge. This bending condition varies based on the screw position and the distance between the two innermost screws.

The desired yield stress is in the range of 400 MPa. The stress variation has to initiate and propagate a crack with the screw and implant being estimated whereas the stress raisers will lead to the implant failure. The standing load itself (applied on the distal extremity) needed to be representative of the minimum strength of the human activity performed by the patient between surgery and implant failure. In addition, implant stress becomes relevant to determine the amount of transfer stress from the implant to the neighbouring bone which may affect the implant stability in terms of micro movement. The presence of the two innermost screws acts as a lever or fulcrum. These changes in distance are known as the ‘working length’. Further factors considered in the internal fixation of bone plate fixation include the implant working length.

### 3.2. Effect of Implant Type (Unicortical) on the Stress Distribution of Screw and LCP Plate

**Figure 8:**
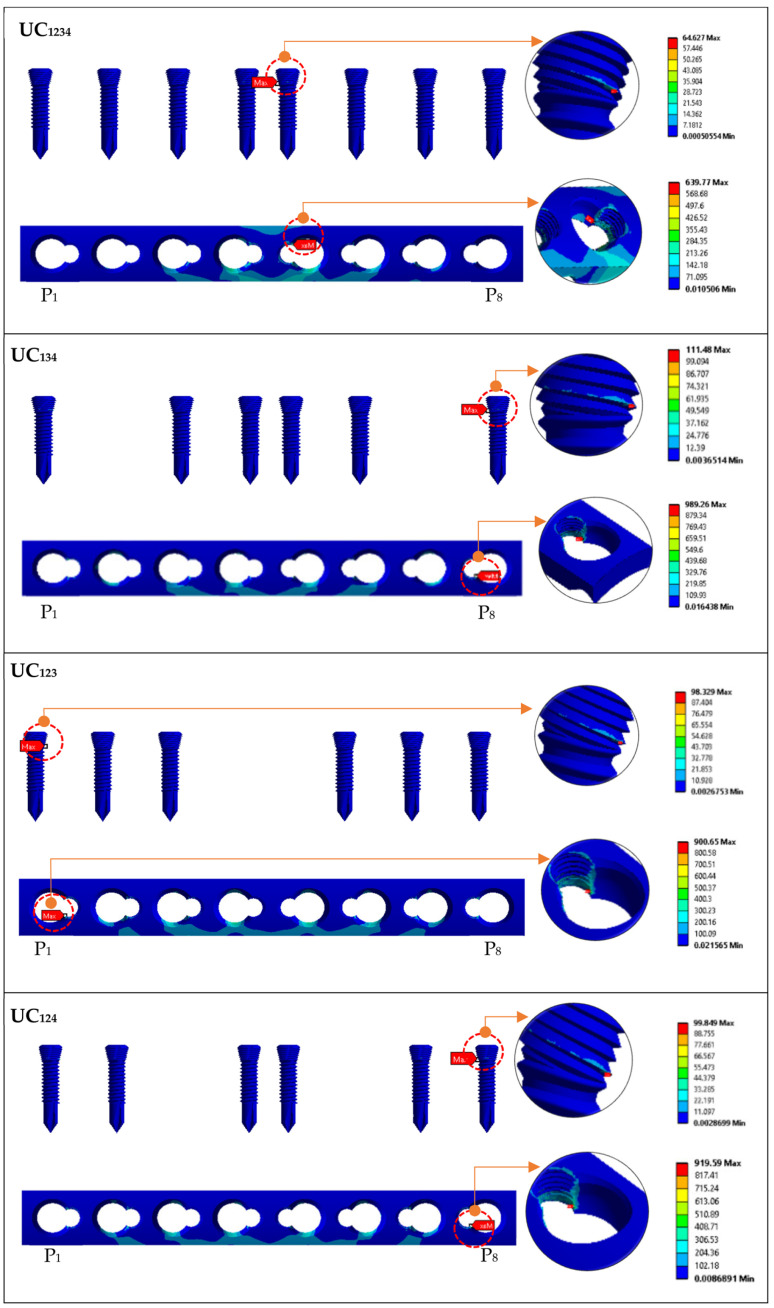
Maximum von Mises stress on implant screws and plate for group UL (locking unicortical) based on standing loading.

Figure 8 shows the maximum von Mises stress on the implant screws and plate for locking the unicortical screw; it had a uniform stress distribution. Configuration UL_1234_ had high stress concentrated at position P5, which focused on the threaded head, while the plate concentrated on the hole threaded for screws and plate. Configuration UL_134_ had high stress concentrated at position P8, which focused on the end of the threaded head, while the plate concentrated on the hole threaded for the screws and plate.

Configuration UL_123_ showed the critical stress concentrated on the P1 screw position, and it was located on the screw head and neck. Similar observations were found at position P1, where critical stress was found in the threaded hole. The failure stress of UL_1234_ was higher than that for UC_1234_, the screw failure was shown based on the type of screw. Stress riser initiates the locking mechanism of the UL screw. Similarly, Configuration UL_124_ showed the critical stress concentrated on the P8 screw position, and it was located on the screw head and neck. Matching to the plate, position P8 indicated critical areas for the fracture of the thread conjunctions and screw holes. The same observations were found in UL_134_ and UL_124_. Overall observations found that the critical failure stress occurred on the screw head with the thread and the screw threaded hole. This was because critical stress transfer occurred as the locking head screw engaged and locked into the threaded plate hole.

### 3.3. Effect of Implant Type (Bicortical) on the Stress Distribution of Screw and LCP Plate

**Figure 9:**
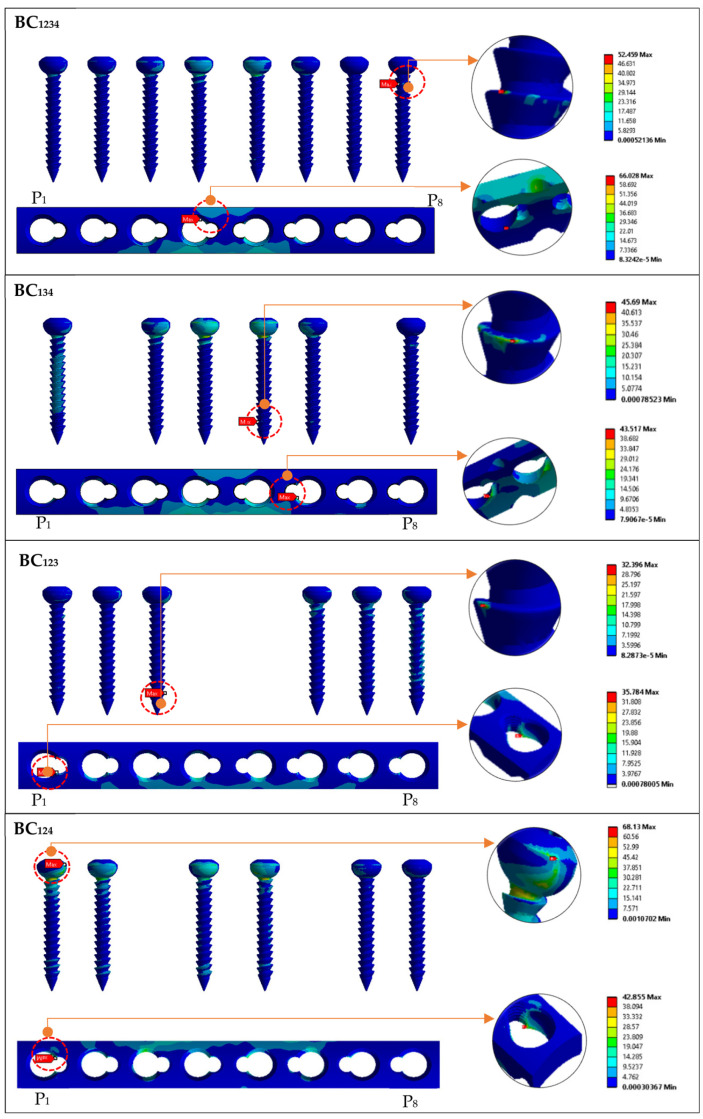
Maximum von Mises stress on implant screws and plate for group BC (bicortical conventional) based on standing loading.

Figure 9 shows the maximum von Mises stress on the implant screws and plate for group BC. Configuration BC_1234_ showed the critical stress concentrated on the screw at position P8, and it was located on the proximal thread. Similar findings were observed for BC_134_ and BC_123_. The results are in good agreement with those reported by Lofaj et al. [14]. Unlike the screws, the plates showed P4 to have critical pressure at the bottom of the plates near the threaded hole. Configuration BC_134_ showed the critical stress concentrated on the position P5 screw position and was located on the screw head and neck. Similar to the plate, position P6 showed a critical area located at the screw hole with thread conjunction and screw holes near the fracture site. Similar plate stress concentrations were also observed for UC_1234_, UC_134_, and UC_123_.

Position P3 of BC_123_ showed that critical stress occurred on the screw and neck. For the plates, the critical stress concentrated on position P6 in the conjunctions of screw holes with thread and screw holes near the fracture site. A similar observation was made for BC_124_, where greater stress was obtained on position P1 for both implants. This is caused by a load transfer from the proximal fragment to the distal fragment through the plate. Most of the critical stress occurred on the distal fragment and concentrated on the screws innermost from the fracture site.

### 3.4. Effect of Working Length as a Function of Screw Configurations on Plate Stress

**Figure 10:**
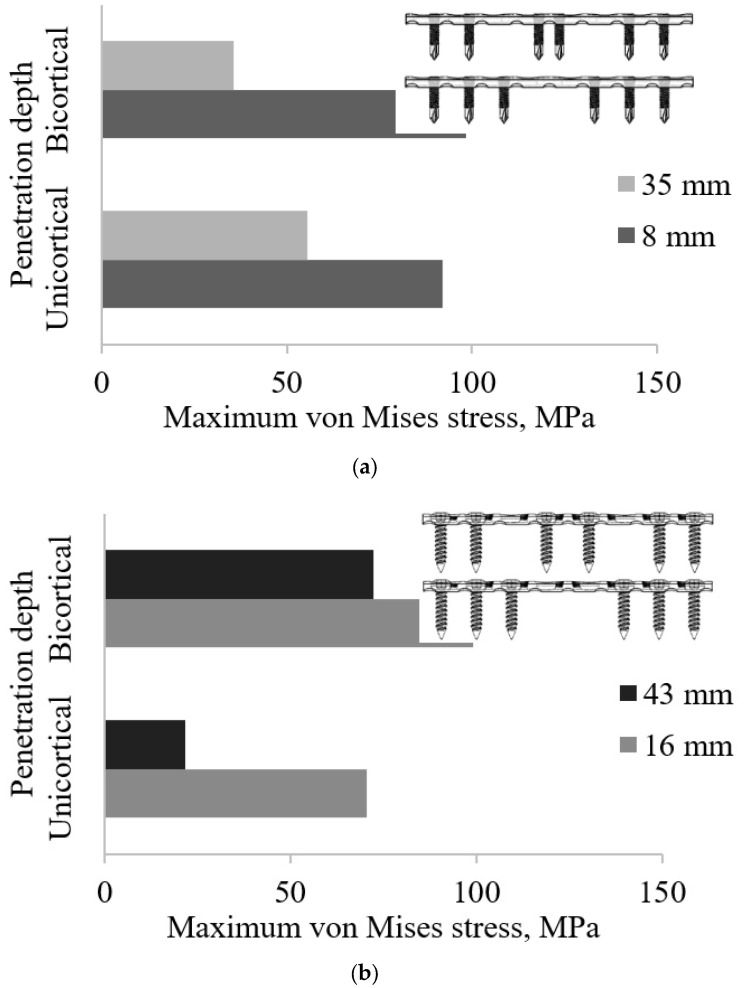
Effect of working length on the plate stress: (**a**) locking screws (Groups UL and BL) and (**b**) conventional screws (Groups UC and BC).

Figure 10a shows the plate strength of the locking screw at different levels of working length. The configurations of the unicortical and bicortical screws for C_1234_, C_134_, and C_124_ generated an 8 mm working length compared with C_123_ of both types of penetration, which provided a 16 mm working length. The working length and screw configuration were more important than the number of screws [42], whereas the rigidity and flexibility of the implant were key to implant stabilisation.

The average of maximum von Mises plate strengths of Groups UL and BL showed that the bicortical screw had more significant changes in plate stress. Increasing the working length from 8 mm to 35 mm reduced the plate stress by 98.33 MPa to 35.78 MPa, respectively. Under axial loading, the reduction for 35 mm was approximately 60% of 8 mm. For the unicortical screw, 8 mm of working length (91.99 MPa) showed a higher stress value compared with 35 mm (55.42 MPa), as shown in Figure 10b. The working length can make the plate overly flexible and can result in plate breakage [47,48].

Figure 10b shows the screw strength of Groups UC and BC at both penetration depths. It seems that the average equivalent stress of 16 mm for the unicortical screw was higher than that of the bicortical screw; however, it was the contrary for 43 mm. As the innermost screws increased from 16 mm to 43 mm, the plate stresses of bicortical (99.24 MPa to 72.3 MPa) and unicortical (70.46 MPa to 21.51 MPa) types were reduced. The pull-out strength variance between unicortical and bicortical screws was between 26% and 44% [49]. Unlike unicortical screws, which experience high stress, it is inversely proportional to the working length. It also provides higher stress on the 16 mm compared with the 43 mm screws, on top of having different stress distributions. In general, both types of screw show that the unicortical was most affected by the plate stress compared with the bicortical screws for the working length range.

The wider working length decreases the equivalent stress in the implant. The working length enables early mobilisation and bone bridges in the fracture gap to occur [50], whereas the small working length minimises plate strain [50]. This relates to the rigidity and flexibility of the implant; however, an unexpected finding was that implant stress was reduced by reducing the working gap of the unicortical locking. The wider working length was found to decrease the plate stress. As expected, reduced plate rigidity increases the εIFM, and thus the bone strain also increased.

### 3.5. Effect of Screw Configuration on the Callus Stress

**Figure 11:**
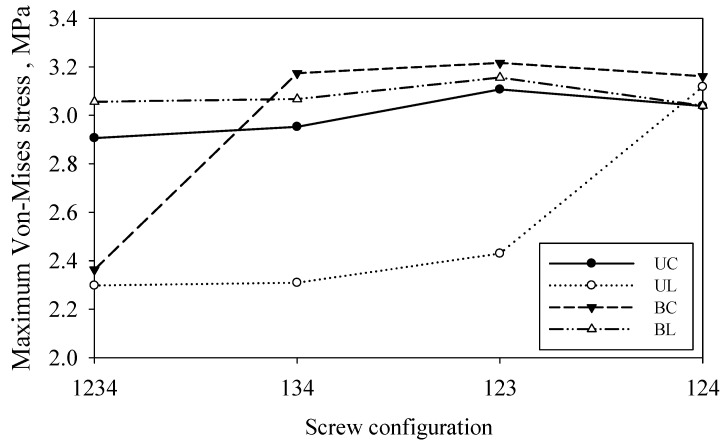
Maximum callus stress under compression load at different screw configurations.

**Figure 12:**
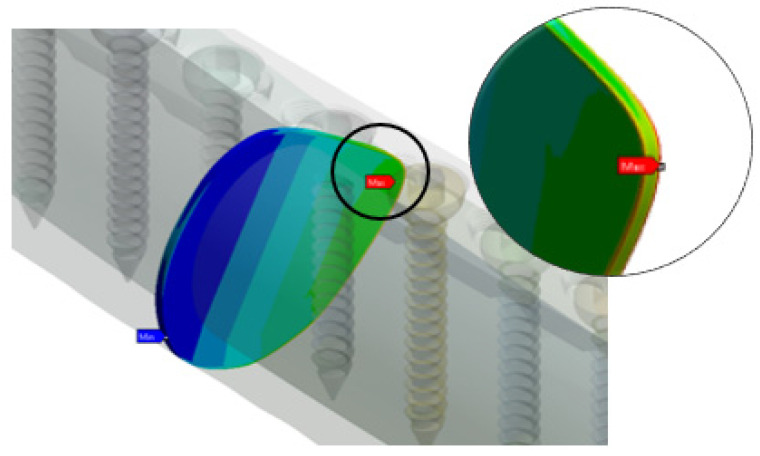
Maximum von Mises stress on the fracture site for different groups of screws.

Figure 11 shows the maximum stress of the callus for both screw types and the penetration depth. Overall, the data show callus stress in the range of approximately 2.2 MPa to 3.2 MPa. The results obtained show good agreement with those reported by Froud et al. They found that the callus stress ranges between 2 MPa and 3 MPa [51]. This supports the hypothesis that bone remodelling after fracture healing is mainly pressure driven with the differentiation of compression structure. It can be correlated between the mechanical strain of callus and bone remodelling and is supported by Wolff’s law [52]. Figure 12 demonstrates that the maximum stress occurred on the fracture site or callus bone at different group screws. Group BL yielded the highest stress for all configuration ranges while group UL had the lowest stress in callus.

### 3.6. Interfragmentary Strain as a Function of Screw Configuration

Table 4 shows the relationship between screw configurations and the interfragmentary strain at a fracture gap with 800 N of compression load. The obtained interfragmentary strain was more significant than 0.01 for all configurations. The correlation between screw configurations and the interfragmentary strain was obtained to examine the implant stability. In a unicortical group, screw configuration had a small and marginal effect on the interfragmentary strain.

In bicortical groups, the amount of strain gradually increased as the pattern of screw configuration changed from C_1234_ to C_124_. Absolute stability was achieved in all configurations of bicortical screws compared with the unicortical group. In addition, relative stability was obtained for both types of unicortical screw groups for all configurations except C_1234_, where it showed a value of less than 2% strain. This result was not reported as a strategy for optimising fixation stability for these types of screws, for screw position relative to the fractured bone.

Compression applied to the fracture produced preloaded continuous contact and, thus, minimised interfragmentary strain. This enabled the osteons to cross the fracture at the compressed surfaces. The osteons may also cross small gaps stabilised by neighbouring compressed areas of contact. However, the minimal strain did not induce the formation of callus. The displacement of the implant micromotion affected the net resorption that begins to bond the gap, while its tension depended on the tolerable limit of soft tissue formed. This can lead to a reduction in the stress-shielding effect. The closer of the innermost screws to the fracture site resulted in a more significant impact on the interfragmentary strain εIFM rather than the number of screws itself. The findings are supported by Lee et al. [49]. They revealed that the appropriate selection of the types of screw and configuration is essential in achieving bone healing, and fewer screws consequently yield less damage to the soft and hard tissues, thus reducing the surgery costs.

### 3.7. Interfragmentary Strain as a Function of Compression Loading

**Figure 13:**
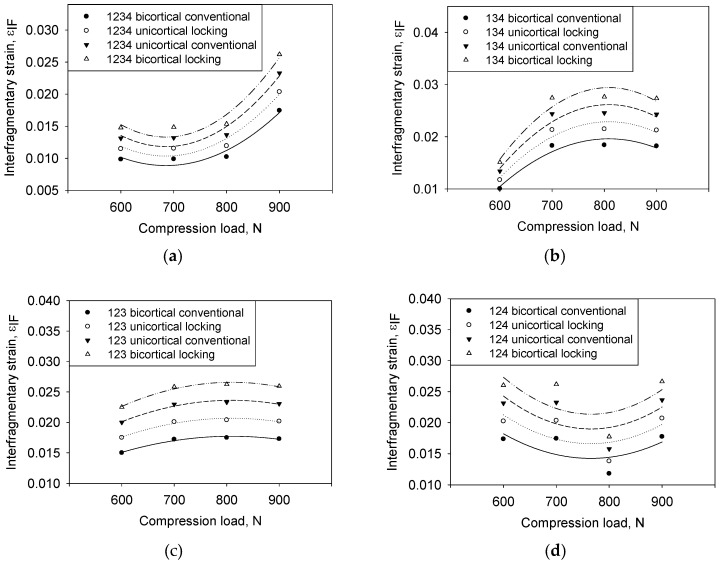
Effect of compression load on the interfragmentary strain at different screw configurations: (**a**) 1234, (**b**) 134, (**c**) 123, and (**d**) 134.

Figure 13 shows the relationship between configurations and interfragmentary strain for all compression loads. In general, the interfragmentary strain increases with increasing loads for all types of screws and configurations. The non-linear regression line was compared to determine the most stable implant of screw type and screw configurations. Figure 13a-d show the responses of εIFM properly fit the curvatures, whereas all data adequately cover the entire range of loads. The effect of increasing loads on the callus strain also gradually increases. However, the effect of changing the screw configuration on load increment cannot be easily summarized. Thus, the correlation of the distribution data was simplified by using regression analysis, as shown in Table 5.

The effects of each type of screw and their configuration were estimated using the residual sum of squares by using 95% of the confidence interval for the parameters. Table 5 shows group BC was the least value for all types of configurations. The small value of the residual sum of squares indicates a more stable implant. The variance shows the effect of εIFM highly depends on the screw type and screw configuration with an increment of loads.

As shown in Table 6, the model specifies the relationship between εIFM and loads, which were compared by the *p*-value obtained from the normality test and shows a significant level fitting to assess the null hypothesis. The screw configuration of C_1234_, C_134_, and C_123_ concluded the model is statistically significant based on non-linear regression as the *p*-value obtained (*p* ≤ 0.05) compared to the configuration 124 (*p* ≥ 0.05). The value of εIFM is statistically significantly different between the screw configurations and compression loads. In these results, the normality of distribution also performed by Shapiro–Wilk indicates a *p*-value > 0.1. Thus, the null hypothesis is accepted for all screw configurations and loads for all input response influence on εIFM.

The constant variance test results show the εIFM passed the test of the assumption that the variance of loads in the screw configuration takes a small variance. The *p*-value computed for the C_1234_ and C_124_ shows an uneven spreading of residual across the fitted line, indicating a non-constant variance as it is far away from a zero value, 0.7048 and 0.9432, respectively.

### 3.8. Interfragmentary Strain as a Function of Compression Loading

**Figure 14:**
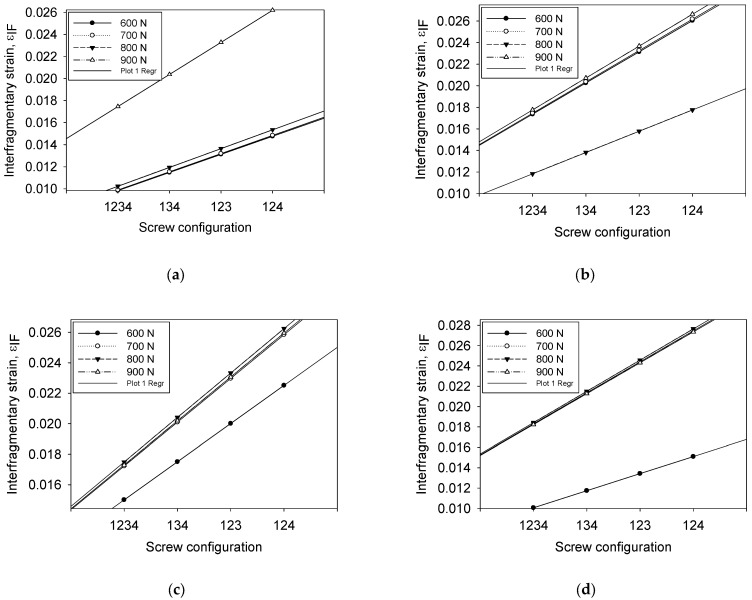
Effect of different screw configurations on interfragmentary strain at different compression loads: (**a**) bicortical conventional, (**b**) bicortical locking, (**c**) unicortical conventional, and (**d**) bicortical locking.

Figure 14 shows a linear relationship between the load and the interfragmentary strain for all different screw configurations. Based on Perren’s theory, the compression load ranges between 700 N and 800 N; this is most critical for changing the tissue phenotype [48]. The increasing pattern of εIFM follows a linear function concerning the compression loading. The steeper slope indicates lesser stability of bone–implant, such as BL compared with group UL, of which the latter is more stable. Researchers have stated that the distance of the fracture gap and loads can quantify the implant stability [50]. However, varying the screw type and configuration also can be the most significant factor contributing to stability. The most stable screw type was group BC, and the most unstable screw type of group UC could be identified as the slope of the regression line, as shown in Figure 14. This result implies that the change in load exertion has a more significant impact on the interfragmentary strain [51] in comparison with changing the screw type.

From the regression analysis, the relationship between interfragmentary strain and compressive load can be linearised as Equation (5):

(5) $$\varepsilon_{IF}=a+bL$$

where *a* and *b* are constants, while L is compression load. The values of *a* and *b* are shown in Equation (2). L is the independent variable, and $\varepsilon_{IF}$ is the dependent variable.

Equation (2) and Table 7 shows that good linear relationships between variables were obtained, as shown in Figure 14a–d. Unexpected, distributed data found in group BC and UC show a high influence on εIFM compared with other groups. The probability of the regression line was used to elucidate the behaviour of variable parameters. This is because the comparison slope of the line can show stability behaviour [52]. At this stage, the stimulation of tissue differentiation during fracture healing after the granulation tissue is initiated [53]. Group BC was less steep (slope = 1.64 × 10^−3^) compared with other configurations, which indicates the least interfragmentary strain and the most-stable construct. It also explains that replacing locked with nonlocked diaphyseal screws does not significantly decrease construct stiffness and does not enhance interfragmentary motion [54].

### 3.9. Correlation between the Controllable Parameters on Interfragmentary Strain as Stability Determination

Table 8 presents the correlation between the screw configuration and screw type. Based on the Pearson correlation analysis, Groups UC and UL have significant values (*p* < 0.01) for 700 N and 800 N of load. The screw configuration for 134 and 124 had a substantial effect on the result of the interfragmentary strain (*p* < 0.05) for both configurations. Moreover, group BC indicated that interfragmentary strain had an effect only for 600 N (*p* < 0.01), and the screw configurations influenced were 1234 and 123 with a significant value (*p* < 0.05). This relationship is related to the location of bone formation and resorption which explained the high and low local mechanical strain [55].

Table 9 shows a correlation between compression load and screw type for the interfragmentary strain. Based on the statistical data, the correlation between 600 N and group BC has a significant effect on the interfragmentary strain. However, an unexpected response was obtained with 700 N and 800 N; this shows the significant impact on the interfragmentary strain (*p* < 0.05), indicating a favourable correlation with screw configuration mainly for group UC (*p* < 0.01). Moreover, the screw configuration under the load is not significant (*p* > 0.05) for 900 N.

## 4. Conclusions

In summary, a bone–implant fixation system consisting of different types of screws with different penetration depths (groups UC, UL, BC, and BL) was developed and investigated. The regression slope of compression was found to be the lowest for group BC (slope = 1.64 × 10^−3^) and highest for group UC, suggesting appropriate sampling distributions for the hypothesis for the strain result. Under compression loading, screw type shows a significant effect (*p* < 0.01) on the interfragmentary strain. The correlation of screw configuration with screw type shows a noteworthy impact on group UC and UL, having a significant value (*p* < 0.01) for 700 N and 800 N of the load and *p* < 0.05 for the configurations of C_134_ and C_124_. The interfragmentary strain had an effect only for 600 N (*p* < 0.01) for group BC and the screw configurations that were influenced were C_1234_ and C_123_ (*p* < 0.05). Moreover, the slope response of ε against the load was steeper, owing to the less stable bone–implant model. The amount of strain optimally ranged between the minimum required for the induction of callus and the maximum that allows bony bridging. Strain values ≤ 2% promote the callus formation and were classified as absolute stability. The correlation between εIFM and the type of screw showed scattered data of low quality for all types of screws mainly for bicortical conventional or group BC. Thus, the obtained determination coefficient was higher to achieve a reliable prediction of stability. If it is desirable to use group BC and C_134_ or BC_134_, it is recommended. This is important to address the biomechanical characteristics of flexible and locked plating to provide a balance between the stability and flexibility of implant fixation constructs and directly promote secondary bone healing. There is an excellent linear correlation between compression loading and screw type. For future research, a similar study can be conducted for different dynamic loading which can medically explain the more actual condition of a fracture. New exploration on the effect of screw configuration at microscale levels that concentrates on the area of the bone–implant interface is highly recommended.

## Figures and Tables

**Table 1:** Statistic mesh node and element of the level of size element for different types of implant.

Type of Configuration	Size Element	Mesh Node	Mesh Element
UC_1234_	0.4	1,749,365	1,030,853
UL_1234_		1,897,652	1,104,719
BC_1234_		1,954,078	1,152,557
BL_1234_		2,171,312	1,272,999
UC_1234_	0.6	975,134	572,176
UL_1234_		1,010,747	582,995
BC_1234_		1,215,571	717,126
BL_1234_		1,448,019	847,188
UC_1234_	0.8	647,338	379,441
UL_1234_		674,924	388,894
BC_1234_		811,360	477,850
BL_1234_		1,054,827	616,522
UC_1234_	1	490,809	290,207
UL_1234_		524,384	129,887
BC_1234_		648,844	385,956
BL_1234_		900,669	527,306

**Table 2:** Type of interaction in the bone–implant model.

Component	Relationship	Remark
Screw–bone (i.e., screw threads)	Rigid (bonded contact)	Fixed all degrees of freedom
Plate–bone	Contact pair	Initially bonded
Fracture surface	Contact pair	Initially bonded
Plate–screw (conventional)	Contact pair	Provide a universal connection between the screw control node and nodes on the bearing surface of the plate.
Plate–screw (locked)	Rigid	Provide a rigid connection between the screw control node and nodes on the bearing surface of the plate.

**Table 3:** Mechanical properties of bone structure and implant materials in FE analysis.

Material	Young’s Modulus, E (GPa)	Poisson’s Ratio (ʋ)	Shear Modulus (GPa)
Trabecular	E_Tb_ = 1.1	ʋ_Tb_ = 0.3	-
Callus	E_callus_ = 0.2	ʋ_callus_ = 0.3	-
Cortical bone *(Longitudinal transverse)*	E_3_ = 20.0	ʋ_12_ = 0.376	G_12_ = 4.53
	E_1_ = 12.0	ʋ_23_ = 0.235	G_23_ = 4.53
	E_2_ = 12.0	ʋ_23_ = 0.376	G_13_ = 4.53
Stainless steel	E_s.s_ = 200	ʋ_S.S_ = 0.3	-

**Table 4:** Effect of screw configuration on the interfragmentary strain of callus at 800 N.

Implant Type	Screw Configuration			
	1234	134	123	124
Group UC	* 0.0175	0.0204	0.0233	0.0262
Group UL	* 0.0184	0.0215	0.0246	0.0276
Group BC	* 0.0102	* 0.0119	* 0.0136	* 0.0154
Group BL	* 0.0118	* 0.0138	* 0.0158	* 0.0178

* absolute stability.

**Table 5:** Residual sum of squares is calculated for each screw design using screw configuration.

Screw Type	Screw Configuration			
	1234	134	123	124
Group UC	3.87 × 10^−6^	5.39 × 10^−6^	2.00 × 10^−7^	2.64 × 10^−5^
Group UL	2.96 × 10^−6^	4.12 × 10^−6^	1.53 × 10^−7^	2.02 × 10^−5^
Group BC	2.18 × 10^−6^	3.03 × 10^−6^	1.12 × 10^−7^	1.49 × 10^−5^
Group BL	4.90 × 10^−6^	6.82× 10^−6^	2.53 × 10^−7^	3.35 × 10^−5^

**Table 6:** Normality analysis on εIFM and the screw configuration.

Screw Configuration	1234	134	123	124
Regression	0.0256	0.0264	0.0003	0.6632
Normality test (Shapiro–Wilk)	* 0.4186	* 0.4186	* 0.4183	* 0.4186
Constant variance test	0.7048	0.0059	0.0072	0.9432
Standard error estimation	0.0019	0.0022	0.0004	0.0049

* Significant level 0.1.

**Table 7:** Slopes εIFM calculated for each screw configuration using compression load.

Implant Type	Compression Load (N)			
	600	700	800	900
Group UC	2.50 × 10^−3^	3.05 × 10^−3^	3.07 × 10^−3^	3.04 × 10^−3^
Group UL	1.68 × 10^−3^	2.91 × 10^−3^	1.97 × 10^−3^	2.96 × 10^−3^
Group BC	1.64 × 10^−3^	1.65 × 10^−3^	1.71 × 10^−3^	2.91 × 10^−3^
Group BL	2.89 × 10^−3^	2.91 × 10^−3^	1.97 × 10^−3^	2.96 × 10^−3^

**Table 8:** Correlations between screw type and interfragmentary strain between regression slopes calculated for compression loads as a control variable.

Screw Type	UC	UL	BC	BL
Correlation	1.000 **	−0.997 **	−0.371	0.338
Significant value	0.000	0.003	0.629	0.662

** Correlation is significant at α = 0.01 level (two-tailed).

**Table 9:** Correlations between compression load and interfragmentary strain between regression slopes calculated for screw type as a control variable.

Compression Load	600 N	700 N	800 N	900 N
Correlation	−0.371	−0.997 **	−1.000 **	0.338
Significant value	0.629	0.003	0.000	0.662

** Correlation is significant at α = 0.01 level (two-tailed).

## Data Availability

The data obtained in this study are available from the corresponding author upon request.
